# Statistical Parametric Mapping in Amyloid Positron Emission Tomography

**DOI:** 10.3389/fnagi.2022.849932

**Published:** 2022-04-25

**Authors:** Natasha M. Smith, Jeremy N. Ford, Arsalan Haghdel, Lidia Glodzik, Yi Li, Debra D’Angelo, Arindam RoyChoudhury, Xiuyuan Wang, Kaj Blennow, Mony J. de Leon, Jana Ivanidze

**Affiliations:** ^1^Department of Radiology and MD Program, Weill Cornell Medicine, New York City, NY, United States; ^2^Department of Radiology, Weill Cornell Medicine, New York City, NY, United States; ^3^Department of Radiology, Massachusetts General Hospital, Boston, MA, United States; ^4^Department of Population Health Sciences, Weill Cornell Medicine, New York City, NY, United States; ^5^Department of Neuroscience and Physiology, University of Gothenburg, Mölndal, Sweden; ^6^Clinical Neurochemistry Laboratory, Sahlgrenska University Hospital, Mölndal, Sweden

**Keywords:** SPM, PET, Alzheimer’s disease, dementia, amyloid

## Abstract

Alzheimer’s disease (AD), the most common cause of dementia, has limited treatment options. Emerging disease modifying therapies are targeted at clearing amyloid-β (Aβ) aggregates and slowing the rate of amyloid deposition. However, amyloid burden is not routinely evaluated quantitatively for purposes of disease progression and treatment response assessment. Statistical Parametric Mapping (SPM) is a technique comparing single-subject Positron Emission Tomography (PET) to a healthy cohort that may improve quantification of amyloid burden and diagnostic performance. While primarily used in 2-[^18^F]-fluoro-2-deoxy-D-glucose (FDG)-PET, SPM’s utility in amyloid PET for AD diagnosis is less established and uncertainty remains regarding optimal normal database construction. Using commercially available SPM software, we created a database of 34 non-*APOE* ε4 carriers with normal cognitive testing (MMSE > 25) and negative cerebrospinal fluid (CSF) AD biomarkers. We compared this database to 115 cognitively normal subjects with variable AD risk factors. We hypothesized that SPM based on our database would identify more positive scans in the test cohort than the qualitatively rated [^11^C]-PiB PET (QR-PiB), that SPM-based interpretation would correlate better with CSF Aβ42 levels than QR-PiB, and that regional z-scores of specific brain regions known to be involved early in AD would be predictive of CSF Aβ42 levels. Fisher’s exact test and the kappa coefficient assessed the agreement between SPM, QR-PiB PET, and CSF biomarkers. Logistic regression determined if the regional z-scores predicted CSF Aβ42 levels. An optimal z-score cutoff was calculated using Youden’s index. We found SPM identified more positive scans than QR-PiB PET (19.1 vs. 9.6%) and that SPM correlated more closely with CSF Aβ42 levels than QR-PiB PET (kappa 0.13 vs. 0.06) indicating that SPM may have higher sensitivity than standard QR-PiB PET images. Regional analysis demonstrated the z-scores of the precuneus, anterior cingulate and posterior cingulate were predictive of CSF Aβ42 levels [OR (95% CI) 2.4 (1.1, 5.1) *p* = 0.024; 1.8 (1.1, 2.8) *p* = 0.020; 1.6 (1.1, 2.5) *p* = 0.026]. This study demonstrates the utility of using SPM with a “true normal” database and suggests that SPM enhances diagnostic performance in AD in the clinical setting through its quantitative approach, which will be increasingly important with future disease-modifying therapies.

## Introduction

Alzheimer’s disease (AD) is the most common cause of dementia worldwide, affecting over five million people in the United States alone. Without disease-modifying therapies, the annual cost to Medicare and Medicaid is expected to surpass $1 trillion by 2050 (Association, 2019). In addition to clinical criteria, cerebrospinal fluid (CSF) biomarkers (Shaw et al., 2018), plasma biomarkers (Zetterberg and Blennow, 2021), and positron emission tomography (PET) with 2-[^18^F]-fluoro-2-deoxy-D-glucose (FDG) have become a cornerstone of AD diagnosis and staging in both research and clinical settings. Specifically, appropriate use of FDG-PET adds clinically valuable information in the diagnostic workup of neurodegenerative disorders and is cost-effective (Silverman et al., 2002), providing a rationale for reimbursement from the Centers for Medicare and Medicaid Services (CMS) (Phurrough et al., 2004).

With the launch of amyloid-targeting therapies such as aducanumab into the clinic, amyloid-targeting PET tracers are poised to have increasing relevance in diagnosis and disease monitoring. At present, despite targeting a principal pathologic substrate, FDA-approved amyloid-binding PET tracers such as [^18^F]-florbetapir, [^18^F]-florbetaben, and [^18^F]-flutemetamol are not typically reimbursed outside of a clinical trial (Cohen et al., 2015), leaving patients to cover the high cost. Another amyloid tracer, [^11^C]-labeled Pittsburgh compound B (PiB), is used almost exclusively in research settings. Notably, PiB was the first widely used amyloid PET tracer and is often used as a reference standard to make comparisons between FDA-approved [^18^F]-labeled amyloid-targeting agents (Landau et al., 2014; Adamczuk et al., 2016; Amadoru et al., 2020). Altogether, amyloid PET agents enhance diagnostic confidence and change management in 20–60% of patients attending memory clinics, guiding decision making on the use of acetylcholinesterase inhibitors (de Wilde et al., 2018; Rabinovici et al., 2019).

In the interpretation of amyloid-targeted PET images, there is significant interrater disagreement with kappa coefficients as low as 0.50 (Lilly, 2013), requiring dedicated training for standardized interpretation (Clark et al., 2011). Amyloid PET scans in normal subjects demonstrate non-amyloid binding in the white matter, possibly to myelin basic protein (Veronese et al., 2015), whereas uptake in neocortical structures is minimal or absent. As validated at post-mortem, in the setting of AD pathology and cortical amyloid-β deposits, there is increased cortical radiotracer avidity, leading to a loss of gray-white matter differentiation on amyloid-targeted cross-sectional PET imaging (Clark et al., 2011). For a standard amyloid PET image, a scan is considered positive if there are: (1) two or more brain areas larger than a single cortical gyrus in which there is reduced or absent gray-white differentiation or (2) one or more areas in which gray matter radioactivity is intense and clearly exceeds amyloid-avidity in adjacent white matter (Lilly, 2013). There is robust sensitivity in the detection of amyloid-β plaques with this standardized interpretation procedure; however, suboptimal interrater variability persists (Clark et al., 2012; Joshi et al., 2012). In the era of amyloid-targeting therapies such as aducanumab, any significant degree of disagreement would be unacceptable given the high cost of the drug and the potential adverse outcome of cerebral edema and hemorrhage (Barakos et al., 2013). Furthermore, this standard qualitative approach provides a binary interpretation which is suboptimal for longitudinal studies and precludes imaging-based treatment response assessment.

Statistical parametric mapping (SPM) is a technique which allows comparison of standardized uptake values (SUVs) within select regions in a given patient to a normal cohort. The technique has been mostly widely and successfully used with FDG-PET (Ishii et al., 2001; Kim et al., 2016). In addition to the quantitative data provided by SPM, semiquantitative data is provided as stereotactic surface projections (SSPs), 3D renderings of the brain showing z-scores as color values. Compared to standard PET images, SPM for FDG-PET has been shown to improve diagnostic performance by multiple groups (Yakushev et al., 2009; Mosconi et al., 2013; Della Rosa et al., 2014; Perani et al., 2014; Ford et al., 2021).

Feasibility of SPM has been demonstrated with [^11^C]-PiB (Ziolko et al., 2006) and more recently with [^18^F]-labeled amyloid-targeting agents (Lilja et al., 2016; Doré et al., 2019; Tanyaluck Thientunyakit et al., 2021). This approach enables the interpreter to quantify amyloid burden rather than offering a binary “positive” or “negative” interpretation. However, direct comparisons of SPM interpretations with standard PET interpretations have been limited. Moreover, a potential pitfall is subject selection for the normal cohort database required for SPM. Up to 30–40% of cognitively normal elderly may have significant amyloid burden on PET, a proportion that is higher among apolipoprotein E (APOE) ε4 carriers (Mielke et al., 2012). The frequency of *APOE* ε4 carriers in the general population is 19.5%, of these, 1.2% are homozygous for ε4 (Jia et al., 2020). A normal database that is enriched with amyloid positive subjects and *APOE* ε4 carriers could diminish sensitivity, risking not identifying patients who could potentially benefit from amyloid-targeting therapeutics.

In addition to FDG and amyloid PET, CSF biomarkers such as total-tau (t-tau), phosphorylated-tau (p-tau), and Aβ42 can provide additional information about disease state and progression. For example, low levels of CSF Aβ42 and high levels of CSF t-tau and p-tau can identify symptomatic AD with a sensitivity and specificity above 80% (Zou et al., 2020). These biomarkers can also be used to identify preclinical AD before symptom onset. Several studies have demonstrated that reduction in CSF Aβ42 levels occur before cognitive decline (Skoog et al., 2003; Gustafson et al., 2007; Stomrud et al., 2007). In a prospective longitudinal study, Bateman et al. found that CSF Aβ42 levels may start to decline 25 years before the onset of AD symptoms, whereas amyloid deposition as measured by [^11^C]-PiB PET and CSF levels of t-tau start to increase 15 years before symptom onset (Bateman et al., 2012). While the CSF levels of Aβ42, t-tau, and p-tau are not a gold standard for predicting or diagnosing AD as other factors may affect the CSF levels of these proteins (Blennow and Hampel, 2003), they are nonetheless a valuable source of information especially in a preclinical, cognitively normal cohort. By using subjects with normal (non-AD range) CSF biomarkers to construct a database, we can be more assured that we have a normal cohort, with low likelihood of having AD-type pathology. Conversely, by obtaining CSF protein levels of a test cohort we can identify cognitively normal individuals at higher risk for developing AD.

Therefore, our purpose was to construct a database of [^11^C]-PiB PET scans from cognitively normal, non-*APOE* ε4 carriers with negative cerebrospinal fluid (CSF) biomarkers for AD and to use SPM on a test cohort of cognitively normal subjects with variable AD risk factors. We hypothesized that SPM based on our database reference cohort would identify more scans as positive in our test cohort than the qualitatively rated [^11^C]-PiB PET (QR-PiB). We further hypothesized that the SPM-based interpretation would correlate better with CSF Aβ42 levels than QR-PiB, and that regional z-scores of specific brain regions known to be involved early in AD, including the precuneus, anterior cingulate, and posterior cingulate would be predictive of CSF Aβ42 levels, whereas the orbitofrontal gyri and middle temporal gyri, similarly sized regions not known to be involved early in AD, would not be predictive.

## Materials and Methods

### Patient Enrollment

**FIGURE 1:**
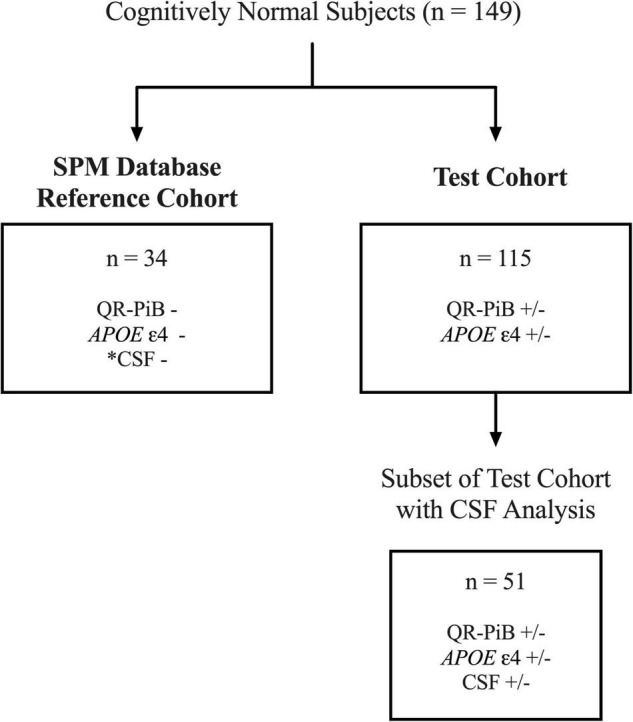
Workflow for the selection of the SPM database reference cohort and test cohort subjects. Of 149 total subjects, 34 cognitively normal, APOE-, QR-PiB-, CSF– subjects were selected to construct the SPM database. *CSF was negative for Aβ42, p-tau, and t-tau biomarkers. The remaining 115 subjects comprise the test cohort. A subset of 51 of the 115 test cohort subjects had CSF available for analysis.

We performed a retrospective analysis of a prospectively enrolled cohort of volunteers who were recruited in IRB approved NIH supported longitudinal studies of normal aging, M. J. de Leon, principal investigator (PI). Subjects were enrolled between 2009 and 2013. Exclusion criteria were mild cognitive impairment, a score > 17 on the 17-item Hamilton Depression Scale, brain tumor, neocortical infarction, and axis I disorders. Of note, a subset of this cohort was previously studied (Glodzik et al., 2014, 2015, 2016; Mosconi et al., 2017, 2018, 2021). After applying exclusion criteria, 149 subjects remained. Subjects were divided into either the SPM database reference cohort (*n* = 34) or the test cohort (*n* = 115). Figure 1 illustrates the workflow of patient selection.

### Imaging: Positron Emission Tomography and Computed Tomography Images

Positron emission tomography (PET)/Computed tomography (CT) images were acquired on General electric (GE) Discovery LS PET/CT and Siemens Biograph PET/CT 64 slice. Two physician-raters with experience in QR-PiB (YL, 13 years of experience; LG, 8 years of experience) provided qualitative interpretations of the QR-PiB images as either positive or negative. The QR-PiB scans were then classified as either positive (both raters identified the scan as positive), indeterminate (only one rater identified the scan as positive), or negative (both raters identified the scan as negative).

### Statistical Parametric Mapping Database Construction

[^11^C]-PiB PET data from PET/CT cases were post-processed using the syngo.via (Siemens Healthineers, Erlangen, Germany) MI Neurology Workflow (*n* = 34). This process involves alignment of PET to the CT using a rigid registration algorithm. Adequate co-registration of PET and anatomical data was confirmed visually by NS, a medical student with four years of neuroradiology research experience and JF, a neuroradiology fellow. To create the atlas, PET images were fit to the Montreal Neurological Institute (MNI) standard space and smoothing was accomplished with an isotropic Gaussian filter of size 12 mm fullwidth at half-maximum. This was followed by intensity normalization to the cerebellum at 75% brightest voxels per the manufacturer’s recommendation in order to mitigate minor imprecisions of region location. A PET template was used to guide the registration of PET data to the MNI geometry. The cerebellum protocol was chosen over automated whole brain because Aβ deposition is low in the cerebellum at all stages of normal and disease (Rowe et al., 2007).

### Statistical Parametric Mapping Analysis

The test cohort (*n* = 115) had both QR-PiB and SPM analysis performed. These two analyses were conducted separately and were blinded to the raters. For the SPM analysis, the images were parsed into Automated Anatomical Labeling (AAL) regions, which have been described previously (Tzourio-Mazoyer et al., 2002). A z-score [(Patient SUV-Healthy Atlas SUV)/Healthy Atlas Standard Deviation] was generated for each AAL region and SSP visualizations with color map gradients were created. The color map provided a visual representation of the number of standard deviations (z-score) each region was above or below the normal database.

Each SSP generated by the SPM analysis was blinded and presented to the same two raters that graded the original QR-PiB scans. Prior to rating, the raters were oriented to the color map and trained on clear examples of positive and negative SSP’s from known AD and non-AD subjects. The SSP’s of the test cohort were graded independently by each rater and assigned a score according to the Likert scale (1 = definitely negative, 2 = probably negative, 3 = indeterminate, 4 = probably positive, 5 = definitely positive). In order to convert the scores to a binary output for comparison against the QR-PiB, the scores from the two raters were averaged and the SSP image was rated as negative (average score < 3), indeterminate (average score = 3), or positive (average score > 3). A subset of the test cohort underwent CSF sampling and analysis (*n* = 51).

### Lumbar Puncture, Cerebrospinal Fluid Collection, and Aβ42 Evaluation

Cerebrospinal fluid was collected by lumbar punctures performed between late morning and early afternoon using a 25-gauge needle guided by fluoroscopy. The CSF samples were centrifuged for 10 min at 1,500 g at 4°C, aliquoted into 0.25 mL polypropylene tubes and then stored at −80°C until assayed. CSF Aβ42 (pg/mL), p-tau (pg/mL), and t-tau (pg/mL) were blindly analyzed in batch mode using enzyme-linked immunosorbent assay (ELISA) (Innotest, Innogenetics/Fujirebio, Ghent, Belgium). The procedures for the lumbar puncture and CSF handling are published (Spiegel et al., 2016). Each CSF biomarker was classified as positive or negative based on the cutoff values as defined by Miners et al. (2019). A positive AD range for each biomarker was defined as Aβ42 < 550 pg/mL, p-tau > 60 pg/mL, or t-tau > 400 pg/mL (Miners et al., 2019).

### Statistical Analysis

Categorical variables are presented as counts or percentages and continuous variables are presented as means ± standard deviations (SD). The Fisher’s exact test was employed to compare the number of patients who received positive, negative, and indeterminate ratings between SPM and QR-PiB alone. Agreement between QR-PiB, SPM and CSF was assessed using the kappa coefficient (κ). In order to assess differences in age and *APOE* ε4 status between those with positive and negative QR-PiB reads, *p*-values were calculated using the unpaired *t*-test for age and two-tailed Fisher exact test for *APOE* ε4 status. Next, a regional analysis was performed on five regions of interest: the anterior cingulate gyrus, the posterior cingulate gyrus, precuneus, middle temporal gyrus, and orbitofrontal gyrus. The anterior cingulate, posterior cingulate, and precuneus were selected given their known early involvement in Aβ deposition (Huang et al., 2013; Grothe et al., 2017; Palmqvist et al., 2017). The middle temporal gyrus and orbitofrontal gyrus, which may be involved in advanced AD, are not known to be specific or early involved regions. A logistic regression model was employed to determine if the z-scores from each studied region predicted a positive rating of the AD biomarker, Aβ42 (<550 pg/mL). For any region whose z-score was a significant predictor of the outcome, an optimal z-score cutoff was calculated using Youden’s index, derived from receiver operating characteristic (ROC) curves, to distinguish positive from negative cases (Supplementary Figure 1).

## Results

### Statistical Parametric Mapping Database

A subset of 34 subjects from the cohort of 149 were selected for construction of the database (Figure 1). In order to be included in the reference database all subjects had to be cognitively normal with a Mini-Mental State Exam (MMSE) ≥ 25, be *APOE* ε4 negative on both alleles, be negative on QR-PiB, and have CSF levels of Aβ42, phosphorylated tau (p-tau), and total-tau (t-tau) in the non-AD range as defined by Miners et al. and described in the Materials and Methods section (Miners et al., 2019). Clinical and demographic characteristics of the SPM database reference cohort are outlined in Table 1.

**TABLE 1:** Cohort characteristics of the statistical parametric mapping (SPM) database.

Number of subjects	34
Gender (M/F)	12/22
Race (White/Non-White)	31/3
Education (years) ± SD	17 ± 1.7
*APOE* ε4 status (−/+)	34/0
Age in years at time of PET scan ± SD	62 ± 7.5
Age in years at time of CSF ± SD	62 ± 7.5
Average time in years between CSF and PET acquisition ± SD	0.25 ± 0.31
MMSE ± SD [Range]	29 ± 1.0 [26–30]
QR-PiB (−/+)	34/0
CSF (−/+)	34/0

### Statistical Parametric Mapping Analysis

The test cohort is comprised of the 115 remaining subjects (Figure 1). None of the subjects used to construct the SPM database were used in the test cohort. Clinical and demographic characteristics of the test cohort are outlined in Table 2. Of the 115 subjects, all had QR-PiB scans and 51 had additional CSF analysis. All patients were cognitively normal with a Mini-Mental State Exam (MMSE) of 29 ± 0.89. Despite all having normal cognition, subjects varied in terms of AD risk factors. 48.7% of subjects were *APOE* ε4 +, 13% had a positive or indeterminate QR-PiB scan, and of the subjects with CSF, 41.2% had Aβ42 levels in the AD range, 27.5% had p-tau levels in the AD range, and 15.6% had t-tau in the AD range (Table 3). *APOE* ε4 positivity was defined as positive for the presence of ε4 on at least one allele (*APOE* X/ε4).

**TABLE 2:** Cohort characteristics of the test group.

Number of subjects	115
Gender (M/F)	36/79
Race (White/Non-White)	99/16
Education (years) ± SD	17 ± 1.9
*APOE* ε4 status (−/+/unknown)	51/56/8
Age in years at time of PET scan ± SD	59.6 ± 11.5
Age in years at time of CSF ± SD	64.4 ± 9.1
Average time in years between CSF and PET acquisition ± SD	0.35 ± 0.42
MMSE ± SD [Range]	29 ± 0.89 [26–30]

**TABLE 3:** Cerebrospinal fluid results of the test group.

CSF (+/−) (*n* = 51)	Aβ42: 21/30 p-tau: 11/40 t-tau: 8/43
CSF Aβ42 pg/mL (avg ± SD)	653 ± 227.4
CSF p-tau pg/mL (avg ± SD)	46 ± 20.1
CSF t-tau pg/mL (avg ± SD)	293 ± 169.8

**FIGURE 2:**
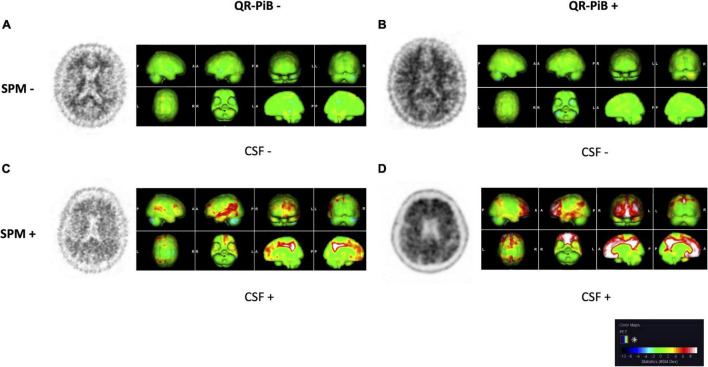
QR-PiB and SPM projections for four cases from the test cohort that represent the spectrum of cases seen in our study. Rows represent SPM− and SPM+ cases, and columns represent QR-PiB− and QR-PiB+ cases. The CSF Aβ42 biomarker status of each case is listed below the SSP. **(A)** A QR-PiB−, SPM−, CSF− subject (69 y/o, M, MMSE: 29, *APOE* ε4−, t-tau = 470 pg/mL, p-tau = 76 pg/mL, Aβ42 = 942 pg/mL). **(B)** A QR-PiB+, SPM−, CSF− subject (58 y/o, F, MMSE: 30, *APOE* ε4−, t-tau = 295 pg/mL, p-tau = 59 pg/mL, Aβ42 = 1,090 pg/mL). **(C)** A QR-PiB−, SPM+, CSF+ subject (73 y/o, M, MMSE: 30, *APOE* ε4+, t-tau = 236 pg/mL, p-tau = 38 pg/mL, Aβ42 = 496 pg/mL). **(D)** A QR-PiB+, SPM+, CSF+ subject (76 y/o, F, MMSE: 29, *APOE* ε4+, t-tau = 705 pg/mL, p-tau = 71 pg/mL, Aβ42 = 465 pg/mL).

Statistical parametric mapping analysis was performed on the test cohort of 115 patients. Representative QR-PiB images and respective SPM maps are shown in Figure 2. Results of the SPM analysis are displayed in Table 4. Use of SPM resulted in statistically significant differences in the categorization of cases. Of note, SPM identified 19.1% of cases as positive while QR-PiB identified only 9.6%. SPM also had a higher proportion of indeterminate cases relative to QR-PiB (7.0 vs. 3.5%).

**TABLE 4:** Diagnostic rating by test type.

Test type	Diagnostic rating			Fisher’s exact test *p*-value
	Positive (*N* = 33)	Indeterminate (*N* = 12)	Negative (*N* = 185)	
SPM	22 (19.1%)	8 (7.0%)	85 (73.9%)	0.002
QR-PiB	11 (9.6%)	4 (3.5%)	100 (87.0%)	

There were eight subjects that were positive on both SPM and QR-PiB. There were 14 subjects who were positive on SPM, but negative or indeterminate on QR-PiB (SPM +, QR-PiB I/−). We found that the SPM +, QR-PiB I/− subjects were older than the average of the entire cohort (*p* = 0.0104) and were more likely to be *APOE* ε4 positive (*p* = 0.0369) (Table 5). The subjects who tested positive on QR-PiB (*n* = 11) were similarly on average older than the average of the entire cohort (*p* = 0.0331), but unlike the SPM positive group were not more likely to be *APOE* ε4 positive (*p* = 0.5219) (Table 6).

**TABLE 5:** Entire test cohort vs. statistical parametric mapping (SPM) positive, QR-PiB indeterminate/negative.

	Entire cohort (*n* = 115)	SPM+, QR-PiB I/− (*n* = 14)	*p*-value
Age in years at time of PET scan ± SD	59.6 ± 11.5	68.18 ± 13.0	0.0104
*APOE* ε4 status (−/+/unknown)	51/56/8	2/11/1	0.0369

**TABLE 6:** Entire test cohort vs. QR-PiB positive.

	Entire cohort (*n* = 115)	QR-PiB + (*n* = 11)	*p*-value
Age in years at time of PET scan ± SD	59.6 ± 11.5	64.86 ± 12.7	0.0331
*APOE* ε4 status (−/+/unknown)	51/56/8	6/4/1	0.5219

**FIGURE 3:**
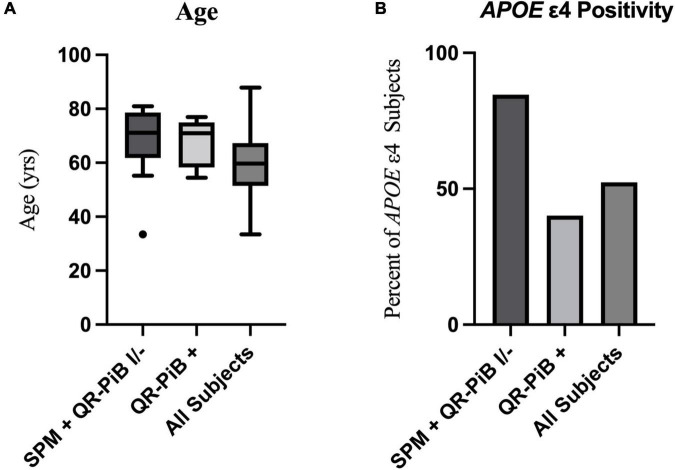
A visual representation of the age distribution with a Tukey box and whisker plot (outlier, black dot) **(A)** and percent of *APOE* ε4 positive subjects **(B)** in the SPM +, QR-PiB I/− subgroup (*n* = 14), the QR-PiB + subgroup (*n* = 11), and the entire test cohort (All Subjects, *n* = 115). There is no significant difference in age between the SPM+, QR-PiB I/− subgroup and the QR-PiB + subgroup **(A)**, but the SPM+, QR-PiB I/− subgroup identifies a higher percentage of *APOE* ε4 positive subjects than the QR-PiB + subgroup **(B)**.

Table 7 demonstrates that the SPM +, QR-PiB I/− subjects (*n* = 14) did not differ significantly in age from the QR-PiB + subjects (*n* = 11) (*p* = 0.8458), but were more likely to be *APOE* ε4 positive (*p* = 0.0393). These results are visually represented in Figure 3.

**TABLE 7:** QR-PiB positive vs. statistical parametric mapping (SPM) positive, QR-PiB indeterminate/negative.

	QR-PiB+ (*n* = 11)	SPM+, QR-PiB I/− (*n* = 14)	*p*-value
Age in years at time of PET scan ± SD	64.86 ± 12.7	68.18 ± 13.0	0.8458
*APOE* ε4 status (−/+/unknown)	6/4/1	2/11/1	0.0393

There were three subjects that were positive on QR-PiB, but negative on SPM. All three subjects had an SPM score of 2.5. Two of the three discordant subjects had CSF; the CSF was negative for all three AD biomarkers (Aβ42, p-tau, and t-tau).

Within the test cohort, a subset of 51 subjects also had CSF. The degree of agreement between SPM and QR-PiB was fair (κ = 0.33). SPM had a stronger correlation with the CSF Aβ42 levels than QR-PiB (κ = 0.13 vs. 0.06) (Table 8).

**TABLE 8:** Diagnostic rating agreement.

Comparison	Kappa coefficient
SPM vs. CSF Aβ42 (P/N)	0.13
QR-PiB vs. CSF Aβ42 (P/N)	0.06
SPM vs. QR-PiB (P/I/N)	0.33

*P = positive, I = indeterminate, N = negative.*

Next, we performed a regional analysis of five regions to determine if any regions were good predictors of the CSF Aβ42 levels (Table 9). The anterior cingulate gyrus [OR: 1.6 (95% CI 1.1, 2.5)], posterior cingulate gyrus [OR: 1.8 (95% CI 1.1, 2.8)], and precuneus [OR: 2.4 (95% CI 1.1, 5.1)], were found to be significant predictors of the CSF Aβ42 levels. The middle orbitofrontal gyrus and middle temporal gyrus were not statistically significant predictors of CSF Aβ42 levels.

**TABLE 9:** Logistic regression using regional Z-score to predict cerebrospinal fluid (CSF) Aβ42 levels (*n* = 51).

Region	OR (95% CI)	*p*-value	Optimal Z-score cut-off
Anterior cingulate gyrus	1.6 (1.1, 2.5)	0.026	2.00
Posterior cingulate gyrus	1.8 (1.1, 2.8)	0.020	0.50
Precuneus	2.4 (1.1, 5.1)	0.024	1.20
Middle frontal gyrus, orbital part	1.3 (1.0, 1.8)	0.084	–
Middle temporal gyrus	1.3 (1.0, 1.8)	0.080	–

## Discussion

We present the construction of a database of cognitively normal subjects who underwent QR-PiB for subsequent application of SPM analysis and direct comparison with QR-PiB interpretation. We were able to construct the database from a cohort of cognitively normal volunteers who had previously been enrolled in a prospective study, and for whom CSF protein data and *APOE* genetics data was also available. With exception of proof-of-concept studies (Ziolko et al., 2006; Lilja et al., 2016; Doré et al., 2019; Tanyaluck Thientunyakit et al., 2021), head-to-head studies comparing amyloid PET SPM to QR-PiB image interpretation have been limited. Two representative studies (Harn et al., 2017; Petrover et al., 2021) used commercially available databases for SPM analysis with [^18^F]-Florbetapir and [^18^F]-Florbetaben, respectively. Both studies demonstrated that SPM improved diagnostic confidence and performance; however, the commercially available databases utilized were comprised of “negative scans” confirmed by expert review without the aid of *APOE* ε4 status and CSF biomarkers. Given the persistent interrater variability among trained interpreters of QR-PiB (Clark et al., 2012; Joshi et al., 2012), it is conceivable that positive scans could be included. Additionally, the fact that CSF biomarker data was not used to construct these databases opens the possibility that subjects with early CSF changes indicative of preclinical AD could be included given that CSF changes can occur up to 25 years before the onset of cognitive symptoms and up to 10 years before QR-PiB changes (Bateman et al., 2012). It is important to note that while positive CSF biomarkers provide valuable adjunct information and convey a degree of increased risk, they are not by themselves diagnostic and as such there is no universally accepted standard for the CSF cutoffs. For the purposes of this study we used the threshold set forth by Miners et al. (2019). Subjects with normal CSF criteria per this threshold were considered to be lower risk for later development of AD.

We used [^11^C]-PiB PET to create and test an amyloid PET database for SPM, using commercially available software (syngo.via MI Neurology analysis tool). A particular advantage of this software over alternatives is that it is clinically integrated which allows for easier clinical translation and has the potential for widespread use. The SPM approach also has the potential to be applied to other PET radiotracers targeting amyloid, tau, or other biomarkers of neurodegeneration.

Our study demonstrates that use of SPM for amyloid-targeted PET may improve sensitivity given that more positive scans were identified relative to interpretation of the standard QR-PiB image (19.1 vs. 9.6%). This invokes the question of whether these represent true or false positives, but given the cross-sectional nature of our study we are not able to identify those individuals who go on to develop AD versus those who do not. Taking this limitation into account, our correlation with CSF biomarkers suggests a greater proportion of cases interpreted as positive are true positives given the greater concordance of SPM with AD biomarkers relative to the QR-PiB interpretation (kappa coefficient 0.13 vs. 0.06). The exact incidence of true versus false positives could be identified with future prospective longitudinal studies. While the kappa coefficient between CSF biomarkers and SPM interpretation appears low, it is important to reiterate that these subjects are cognitively normal, and if indeed there is AD pathology, they would be early in the disease process. Among cognitively normal, amnestic MCI, and AD subjects, it has been shown that CSF Aβ42 does not correlate with cognitive scores (Vemuri et al., 2009). Moreover, AD-type changes in CSF may be a feature of normal aging (Toledo et al., 2015) and may be present in up to one-third of cognitively normal subjects (De Meyer et al., 2010).

Of note, there was a higher proportion of indeterminate cases with the use of SPM (7.0 vs. 3.5%). One potential explanation for this is that the raters had never used SPM before to determine amyloid status and received limited training, whereas the raters were well-versed in the interpretation of QR-PiB. Therefore, additional training in SPM interpretation is likely to increase rater confidence, similar to the effect that training had on standard PET interpretation (Clark et al., 2011). The reader unfamiliarity with interpreting SPM for amyloid PET and the subsequent higher number of indeterminate cases likely contributed to the low kappa coefficient representing the correlation between the SPM read and the CSF Aβ42 levels. Further training with SPM and validation of quantifiable z-score cutoffs could serve to further increase the correlation. Nonetheless, SPM-naïve raters still identified more positive cases with the use of SPM.

There were three cases that were QR-PiB positive, SPM negative. In total, these represent only 2.6% of the entire test cohort. A study by Ikonomovic et al. (2018) found the rate of false positives on amyloid PET, even among experienced raters such as ours, to be 2.8%. In addition, the lack of CSF amyloid biomarkers favors the qualitative reads to have been false positives, possibly due to poor scan quality, scan timing, or cortical atrophy (Apostolova et al., 2016).

When we further examined the cases that were identified as positive on SPM, but negative or indeterminate on QR-PiB we found that SPM, similar to QR-PiB, identified individuals whose average age was significantly higher than that of the entire cohort (SPM *p* = 0.0104, QR-PiB *p* = 0.0331). Given that amyloid deposition increases with age, this is in line with what would be expected (Mormino and Papp, 2018; Zhang et al., 2021). Interestingly, SPM was able to identify a higher proportion of *APOE* ε4 positive individuals (*p* = 0.0369) than were present in the entire cohort whereas QR-PiB did not identify more *APOE* ε4 positive subjects (*p* = 0.5219). This indicates SPM may be able to identify *APOE* ε4 subjects with increased sensitivity over QR-PiB.

Future work will focus on establishing region-specific z-score cutoffs. Certain regions such as the precuneus and posterior cingulate are known to have early accumulation of Aβ42 (Karas et al., 2007; Grothe et al., 2017; Mi et al., 2017; Palmqvist et al., 2017). Palmqvist et al. demonstrated that 15 of 68 cortical regions including the precuneus, anterior cingulate, and posterior cingulate had an increased rate of amyloid accumulation in cognitively normal subjects with low levels of CSF Aβ42 (CSF+) but negative ^18^F-florbetapir PET scans (PET-) indicating these regions accumulate Aβ early in the disease process (Bateman et al., 2012; Palmqvist et al., 2017). As such, global z-score analysis could underemphasize the pivotal role that select regions may have in portending progression of AD.

Even in the absence of z-score cutoffs, visual interpretation of SPM SSPs alone provides improvements in diagnostic performance relative to interpretation of preprocessed PET images. This has been well-validated with the use of FDG-PET for dementia diagnosis (Perani et al., 2014). Perani et al. demonstrated that visual interpretation of SSPs rather than standard FDG-PET improved sensitivity for the detection of neurodegenerative disorders from 78 to 96% and increased specificity from 50 to 84%. The interpretation of SSP maps from FDG-PET in conjunction with z-scores reflects how FDG-PET SPM is currently used in clinical practice, and we foresee a similar role for SPM in amyloid PET.

While we were able to identify region specific cutoffs for our dataset, these specific cutoffs may not be generalizable given that our population was largely cognitively normal (MMSE ≥ 25). In the clinical setting, amyloid PET scans are ordered in patients who usually demonstrate cognitive impairment. A Cochrane review on FDG-PET for dementia highlights that it has been challenging to establish thresholds given the heterogeneity of study populations (Morbelli et al., 2015). Nonetheless, we believe greater standardization is achievable with amyloid PET, and hope to explore valid, region-specific z-score thresholds in future studies of clinical populations.

In our region-specific analysis, the precuneus had the highest odds-ratio in predicting CSF Aβ42 levels. While this data was collected from a single timepoint, it suggests early involvement of the precuneus in AD pathology, concordant with other studies demonstrating early precuneus involvement in AD (Karas et al., 2007; Mi et al., 2017). Interestingly, the anterior cingulate demonstrated a higher odds ratio than the posterior cingulate, which is seemingly discordant from FDG-PET related findings (Minoshima et al., 1997; Brown et al., 2014; Ford et al., 2021). However, there is evidence that amyloid deposits may occur early in the anterior cingulate in AD (Grothe et al., 2017; Palmqvist et al., 2017). Future longitudinal studies in cognitively normal individuals using our SPM database could further illuminate the sequence of amyloid deposition in preclinical AD and normal aging.

There are several notable limitations of this study. There is lack of diversity in the enrolled population this retrospective analysis was based on, with the cohort of volunteers primarily comprised of persons identifying as Caucasian; enrollment of a diverse population reflecting the population affected by cognitive impairment and dementia will be prioritized in future prospective studies. Moreover, the mean age of the normal database was 62 ± 7.5 years, which may be less relevant for elderly patients (e.g., >80 years old). Furthermore, the CSF biomarkers applied here are somewhat inadequate given that specificities and positive predictive values are insufficient to diagnose AD prior to meeting clinical criteria (Hertze et al., 2010). Also, as mentioned, AD-type CSF biomarkers (De Meyer et al., 2010; Toledo et al., 2015) and amyloid deposition (Mielke et al., 2012) may be expected findings of normal aging. Longitudinal assessment with SPM would be the most suitable approach to confirm which region-based z-score cutoffs are clinically relevant and predictive of future conversion to AD. Another noteworthy limitation is that the amyloid PET scans were acquired from 2009 to 2013, and there have since been improvements on multiple fronts in amyloid PET, including motion correction (Brendel et al., 2015) as well as the utilization of low-dose technique in conjunction with deep learning (Chen et al., 2019). As such, to ensure accuracy of SPM analysis, future prospective studies will require the construction of new reference databases to match acquisition and post-processing protocols that incorporate these technical advances.

Emerging therapeutic options for AD, including amyloid targeting antibodies such as aducanumab (Cummings et al., 2021), underscore the critical need for early and accurate identification of AD pathology. Amyloid targeted PET with SPM analysis can take PET interpretation from a primarily qualitative to a quantitative approach, allowing more accurate quantification of cortical amyloid burden, and laying the basis for longitudinal follow up in the context of treatment response assessment. With further validation, SPM with amyloid PET may become routine in clinical practice, analogous to FDG PET for dementia evaluation. Given the high-cost of current and future amyloid-modulating therapies, and given ongoing considerations by the Centers for Medicare and Medicaid Services to lift PET payment restrictions (Stempniak, 2021), it is conceivable that amyloid PET with SPM will eventually become reimbursable from third party payers, thereby increasing access to this important diagnostic tool within the population-at-large.

## Data Availability Statement

The raw data supporting the conclusions of this article will be made available by the authors, without undue reservation.

## Ethics Statement

The studies involving human participants were reviewed and approved by Weill Cornell Medicine IRB. The patients/participants provided their written informed consent to participate in this study.

## Author Contributions

JI, NS, and JF: conceptualization and writing—original draft. MdL: participant enrollment, PET image acquisition, and CSF collection. KB: CSF protein analysis. NS and XW: data curation. YL and LG: qualitative PET review and scoring and SPM image review and scoring. NS, JF, AH, and JI: SPM database construction. AH, NS, and JF: SPM analysis of test cohort. DD’A, AR, and NS: statistical analysis. All authors: writing—review and editing and approved the submitted version.

## Conflict of Interest

The authors declare that the research was conducted in the absence of any commercial or financial relationships that could be construed as a potential conflict of interest.

## Publisher’s Note

All claims expressed in this article are solely those of the authors and do not necessarily represent those of their affiliated organizations, or those of the publisher, the editors and the reviewers. Any product that may be evaluated in this article, or claim that may be made by its manufacturer, is not guaranteed or endorsed by the publisher.

## Supplementary Material

Supplementary Figure 1 Receiver operating characteristic (ROC) curves generated for the anterior cingulate **(A)**, posterior cingulate **(B)**, and precuneus **(C)** regions. An optimal z-score cutoff for each region was calculated using Youden’s index.

## References

[B1] Adamczuk K. Schaeverbeke J. Nelissen N. Neyens V. Vandenbulcke M. Goffin K. (2016). Amyloid imaging in cognitively normal older adults: comparison between 18 F-flutemetamol and 11 C-Pittsburgh compound B. *Eur. J. Nuclear Med. Mol. Imag.* 43 142–151. 10.1007/s00259-015-3156-9 26260650

[B2] Amadoru S. Doré V. Mclean C. A. Hinton F. Shepherd C. E. Halliday G. M. (2020). Comparison of amyloid PET measured in Centiloid units with neuropathological findings in Alzheimer’s disease. *Alzheimer’s Res. Ther.* 12:22. 10.1186/s13195-020-00587-5 32131891 PMC7057642

[B3] Apostolova L. G. Haider J. M. Goukasian N. Rabinovici G. D. Chételat G. Ringman J. M. (2016). Critical review of the Appropriate Use Criteria for amyloid imaging: effect on diagnosis and patient care. *Alzheimers Dement* 5 15–22. 10.1016/j.dadm.2016.12.001 28054024 PMC5198877

[B4] Association A. S. (2019). 2019 Alzheimer’s disease facts and figures. *Alzheimer’s Dement.* 15 321–387. 10.1016/j.jalz.2019.01.010

[B5] Barakos J. Sperling R. Salloway S. Jack C. Gass A. Fiebach J. (2013). MR imaging features of amyloid-related imaging abnormalities. *Am. J. Neuroradiol.* 34 1958–1965. 10.3174/ajnr.A3500 23578674 PMC7965435

[B6] Bateman R. J. Xiong C. Benzinger T. L. Fagan A. M. Goate A. Fox N. C. (2012). Clinical and biomarker changes in dominantly inherited Alzheimer’s disease. *N Engl. J. Med.* 367 795–804. 10.1056/NEJMoa1202753 22784036 PMC3474597

[B7] Blennow K. Hampel H. (2003). CSF markers for incipient Alzheimer’s disease. *Lancet Neurol.* 2 605–613. 10.1016/s1474-4422(03)00530-1 14505582

[B8] Brendel M. Högenauer M. Delker A. Sauerbeck J. Bartenstein P. Seibyl J. (2015). Improved longitudinal [18F]-AV45 amyloid PET by white matter reference and VOI-based partial volume effect correction. *Neuroimage* 108 450–459. 10.1016/j.neuroimage.2014.11.055 25482269

[B9] Brown R. K. Bohnen N. I. Wong K. K. Minoshima S. Frey K. A. (2014). Brain PET in suspected dementia: patterns of altered FDG metabolism. *Radiographics* 34 684–701. 10.1148/rg.343135065 24819789

[B10] Chen K. T. Gong E. De Carvalho Macruz F. B. Xu J. Boumis A. Khalighi M. (2019). Ultra–low-dose 18F-florbetaben amyloid PET imaging using deep learning with multi-contrast MRI inputs. *Radiology* 290 649–656. 10.1148/radiol.2018180940 30526350 PMC6394782

[B11] Clark C. M. Mintun M. A. Pontecorvo M. J. (2011). Florbetapir-PET Imaging and Postmortem β-Amyloid Pathology—Reply. *Jama* 305 1857–1858. 10.1001/jama.2011.579 21558513

[B12] Clark C. M. Pontecorvo M. J. Beach T. G. Bedell B. J. Coleman R. E. Doraiswamy P. M. (2012). Cerebral PET with florbetapir compared with neuropathology at autopsy for detection of neuritic amyloid-β plaques: a prospective cohort study. *Lancet Neurol.* 11 669–678. 10.1016/S1474-4422(12)70142-4 22749065

[B13] Cohen J. P. Dong J. Lu C. Y. Chakravarthy R. (2015). Restricting access to florbetapir: medicare coverage criteria for diagnostics and drugs are inconsistent. *BMJ* 351:h3333. 10.1136/bmj.h3333 26141315

[B14] Cummings J. Aisen P. Apostolova L. G. Atri A. Salloway S. Weiner M. (2021). Aducanumab: Appropriate Use Recommendations. *J. Prev. Alzheimers Dis.* 8 398–410. 34585212 10.14283/jpad.2021.41 PMC8835345

[B15] De Meyer G. Shapiro F. Vanderstichele H. Vanmechelen E. Engelborghs S. De Deyn P. P. (2010). Diagnosis-independent Alzheimer disease biomarker signature in cognitively normal elderly people. *Arch. Neurol.* 67 949–956. 10.1001/archneurol.2010.179 20697045 PMC2963067

[B16] de Wilde A. Van Der Flier W. M. Pelkmans W. Bouwman F. Verwer J. Groot C. (2018). Association of amyloid positron emission tomography with changes in diagnosis and patient treatment in an unselected memory clinic cohort: the ABIDE project. *JAMA Neurol.* 75 1062–1070. 10.1001/jamaneurol.2018.1346 29889941 PMC6143118

[B17] Della Rosa P. A. Cerami C. Gallivanone F. Prestia A. Caroli A. Castiglioni I. (2014). A standardized [18F]-FDG-PET template for spatial normalization in statistical parametric mapping of dementia. *Neuroinformatics* 12 575–593. 10.1007/s12021-014-9235-4 24952892

[B18] Doré V. Bullich S. Rowe C. C. Bourgeat P. Konate S. Sabri O. (2019). Comparison of 18F-florbetaben quantification results using the standard Centiloid, MR-based, and MR-less CapAIBL§approaches: validation against histopathology. *Alzheimer’s Dement.* 15 807–816. 10.1016/j.jalz.2019.02.005 31101517

[B19] Ford J. N. Sweeney E. M. Skafida M. Glynn S. Amoashiy M. Lange D. J. (2021). Heuristic scoring method utilizing FDG-PET statistical parametric mapping in the evaluation of suspected Alzheimer disease and frontotemporal lobar degeneration. *Am. J. Nucl. Med. Mol. Imag.* 11 313–326. 34513285 PMC8414399

[B20] Glodzik L. Kuceyeski A. Rusinek H. Tsui W. Mosconi L. Li Y. (2014). Reduced glucose uptake and Aβ in brain regions with hyperintensities in connected white matter. *Neuroimage* 100 684–691. 10.1016/j.neuroimage.2014.06.060 24999038 PMC4138232

[B21] Glodzik L. Rusinek H. Kamer A. Pirraglia E. Tsui W. Mosconi L. (2016). Effects of vascular risk factors, statins, and antihypertensive drugs on PiB deposition in cognitively normal subjects. *Alzheimers Dement* 2 95–104. 10.1016/j.dadm.2016.02.007 27239540 PMC4879519

[B22] Glodzik L. Rusinek H. Li J. Zhou C. Tsui W. Mosconi L. (2015). Reduced retention of Pittsburgh compound B in white matter lesions. *Eur. J. Nucl. Med. Mol. Imag.* 42 97–102. 10.1007/s00259-014-2897-1 25331458 PMC4415610

[B23] Grothe M. J. Barthel H. Sepulcre J. Dyrba M. Sabri O. Teipel S. J. (2017). In vivo staging of regional amyloid deposition. *Neurology* 89 2031–2038. 10.1212/wnl.0000000000004643 29046362 PMC5711511

[B24] Gustafson D. R. Skoog I. Rosengren L. Zetterberg H. Blennow K. (2007). Cerebrospinal fluid beta-amyloid 1-42 concentration may predict cognitive decline in older women. *J. Neurol. Neurosurg. Psychiatry* 78 461–464. 10.1136/jnnp.2006.100529 17098843 PMC2117838

[B25] Harn N. R. Hunt S. L. Hill J. Vidoni E. Perry M. Burns J. M. (2017). Augmenting amyloid PET interpretations with quantitative information improves consistency of early amyloid detection. *Clin. Nuclear Med.* 42 577–581. 10.1097/RLU.0000000000001693 28574875 PMC5491352

[B26] Hertze J. Minthon L. Zetterberg H. Vanmechelen E. Blennow K. Hansson O. (2010). Evaluation of CSF biomarkers as predictors of Alzheimer’s disease: a clinical follow-up study of 4.7 years. *J. Alzheimer’s Dis.* 21 1119–1128. 10.3233/jad-2010-100207 21504133

[B27] Huang K.-L. Lin K.-J. Hsiao I.-T. Kuo H.-C. Hsu W.-C. Chuang W.-L. (2013). Regional amyloid deposition in amnestic mild cognitive impairment and Alzheimer’s disease evaluated by [18F] AV-45 positron emission tomography in Chinese population. *PLoS One* 8:e58974. 10.1371/journal.pone.0058974 PMC3597555 23516589

[B28] Ikonomovic M. D. Fantoni E. R. Farrar G. Salloway S. (2018). Infrequent false positive [(18)F]flutemetamol PET signal is resolved by combined histological assessment of neuritic and diffuse plaques. *Alzheimers Res. Ther.* 10:60. 10.1186/s13195-018-0387-6 29935545 PMC6015459

[B29] Ishii K. Willoch F. Minoshima S. Drzezga A. Ficaro E. P. Cross D. J. (2001). Statistical brain mapping of 18F-FDG PET in Alzheimer’s disease: validation of anatomic standardization for atrophied brains. *J. Nucl. Med.* 42 548–557. 11337540

[B30] Jia L. Xu H. Chen S. Wang X. Yang J. Gong M. (2020). The APOE ε4 exerts differential effects on familial and other subtypes of Alzheimer’s disease. *Alzheimers Dement* 16 1613–1623. 10.1002/alz.12153 32881347 PMC7984370

[B31] Joshi A. D. Pontecorvo M. J. Clark C. M. Carpenter A. P. Jennings D. L. Sadowsky C. H. (2012). Performance characteristics of amyloid PET with florbetapir F 18 in patients with Alzheimer’s disease and cognitively normal subjects. *J. Nuclear Med.* 53 378–384. 10.2967/jnumed.111.090340 22331215

[B32] Karas G. Scheltens P. Rombouts S. Van Schijndel R. Klein M. Jones B. (2007). Precuneus atrophy in early-onset Alzheimer’s disease: a morphometric structural MRI study. *Neuroradiology* 49 967–976. 10.1007/s00234-007-0269-2 17955233

[B33] Kim J. Cho S. G. Song M. Kang S. R. Kwon S. Y. Choi K. H. (2016). Usefulness of 3-dimensional stereotactic surface projection FDG PET images for the diagnosis of dementia. *Medicine* 95:e5622. 10.1097/MD.0000000000005622 27930593 PMC5266065

[B34] Landau S. Thomas B. Thurfjell L. Schmidt M. Margolin R. Mintun M. (2014). Amyloid PET imaging in Alzheimer’s disease: a comparison of three radiotracers. *Eur. J. Nuclear Med. Mol. Imag.* 41 1398–1407. 10.1007/s00259-014-2753-3 24647577 PMC4055504

[B35] Lilja J. Thurfjell L. Sörensen J. (2016). Visualization and quantification of 3-dimensional stereotactic surface projections for 18F-Flutemetamol PET using variable depth. *J. Nuclear Med.* 57 1078–1083. 10.2967/jnumed.115.169169 26912445

[B36] Lilly E. (2013). *Package Insert: Florbetapir.* Available online at: https://pi.lilly.com/us/amyvid-uspi.pdf [accessed on Nov 12, 2021].

[B37] Stempniak M. (2021). *Imaging Advocate Applauds CMS’ Decision to Lift Longstanding PET Payment Restriction. Radiology Business.* Available online at: https://www.radiologybusiness.com/topics/economics/imaging-cms-decision-pet-payment-restriction-oncology [accessed on Dec 18, 2021]

[B38] Mi Z. Abrahamson E. E. Ryu A. Y. Fish K. N. Sweet R. A. Mufson E. J. (2017). Loss of precuneus dendritic spines immunopositive for spinophilin is related to cognitive impairment in early Alzheimer’s disease. *Neurobiol. Aging* 55 159–166. 10.1016/j.neurobiolaging.2017.01.022 28259365 PMC5440205

[B39] Mielke M. M. Wiste H. J. Weigand S. D. Knopman D. S. Lowe V. J. Roberts R. O. (2012). Indicators of amyloid burden in a population-based study of cognitively normal elderly. *Neurology* 79 1570–1577. 10.1212/WNL.0b013e31826e2696 22972644 PMC3475629

[B40] Miners J. S. Kehoe P. G. Love S. Zetterberg H. Blennow K. (2019). CSF evidence of pericyte damage in Alzheimer’s disease is associated with markers of blood-brain barrier dysfunction and disease pathology. *Alzheimers Res. Ther.* 11:81. 10.1186/s13195-019-0534-8 31521199 PMC6745071

[B41] Minoshima S. Giordani B. Berent S. Frey K. A. Foster N. L. Kuhl D. E. (1997). Metabolic reduction in the posterior cingulate cortex in very early Alzheimer’s disease. *Ann. Neurol.* 42 85–94. 10.1002/ana.410420114 9225689

[B42] Morbelli S. Garibotto V. Van De Giessen E. Arbizu J. Chételat G. Drezgza A. (2015). A Cochrane review on brain [^18^F]FDG PET in dementia: limitations and future perspectives. *Eur. J. Nucl. Med. Mol. Imag.* 42 1487–1491. 10.1007/s00259-015-3098-2 26067090

[B43] Mormino E. C. Papp K. V. (2018). Amyloid Accumulation and Cognitive Decline in Clinically Normal Older Individuals: Implications for Aging and Early Alzheimer’s Disease. *J. Alzheimers Dis.* 64 S633–S646. 10.3233/JAD-179928 29782318 PMC6387885

[B44] Mosconi L. Berti V. Dyke J. Schelbaum E. Jett S. Loughlin L. (2021). Menopause impacts human brain structure, connectivity, energy metabolism, and amyloid-beta deposition. *Sci. Rep.* 11:10867. 10.1038/s41598-021-90084-y 34108509 PMC8190071

[B45] Mosconi L. Berti V. Quinn C. Mchugh P. Petrongolo G. Varsavsky I. (2017). Sex differences in Alzheimer risk: brain imaging of endocrine vs chronologic aging. *Neurology* 89 1382–1390. 10.1212/WNL.0000000000004425 28855400 PMC5652968

[B46] Mosconi L. Rahman A. Diaz I. Wu X. Scheyer O. Hristov H. W. (2018). Increased Alzheimer’s risk during the menopause transition: a 3-year longitudinal brain imaging study. *PLoS One* 13:e0207885. 10.1371/journal.pone.0207885 PMC6291073 30540774

[B47] Mosconi L. Rinne J. O. Tsui W. H. Murray J. Li Y. Glodzik L. (2013). Amyloid and metabolic positron emission tomography imaging of cognitively normal adults with Alzheimer’s parents. *Neurobiol. Aging* 34 22–34. 10.1016/j.neurobiolaging.2012.03.002 22503001 PMC3402654

[B48] Palmqvist S. Schöll M. Strandberg O. Mattsson N. Stomrud E. Zetterberg H. (2017). Earliest accumulation of β-amyloid occurs within the default-mode network and concurrently affects brain connectivity. *Nat. Commun.* 8:1214. 10.1038/s41467-017-01150-x 29089479 PMC5663717

[B49] Perani D. Della Rosa P. A. Cerami C. Gallivanone F. Fallanca F. Vanoli E. G. (2014). Validation of an optimized SPM procedure for FDG-PET in dementia diagnosis in a clinical setting. *Neuroimage Clin.* 6 445–454. 10.1016/j.nicl.2014.10.009 25389519 PMC4225527

[B50] Petrover D. Giliberto L. Clouston S. Gordon M. Franceschi A. (2021). Semiquantitative Approach to Amyloid PET Interpretation in Clinical Practice. *Soc. Nuclear Med.* 62:1068. 10.1055/s-0042-1757290 PMC10010866 36923983

[B51] Phurrough S. Salive M. Richardson S. Cano C. (2004). *Decision Memo for Positron Emission Tomography (FDG) and Other Neuroimaging Devices for Suspected Dementia.* Baltimore: Centers for Medicare & Medicaid Services.

[B52] Rabinovici G. D. Gatsonis C. Apgar C. Chaudhary K. Gareen I. Hanna L. (2019). Association of amyloid positron emission tomography with subsequent change in clinical management among Medicare beneficiaries with mild cognitive impairment or dementia. *Jama* 321 1286–1294. 10.1001/jama.2019.2000 30938796 PMC6450276

[B53] Rowe C. C. Ng S. Ackermann U. Gong S. J. Pike K. Savage G. (2007). Imaging beta-amyloid burden in aging and dementia. *Neurology* 68 1718–1725. 17502554 10.1212/01.wnl.0000261919.22630.ea

[B54] Shaw L. M. Arias J. Blennow K. Galasko D. Molinuevo J. L. Salloway S. (2018). Appropriate use criteria for lumbar puncture and cerebrospinal fluid testing in the diagnosis of Alzheimer’s disease. *Alzheimers Dement* 14 1505–1521. 10.1016/j.jalz.2018.07.220 30316776 PMC10013957

[B55] Silverman D. H. Gambhir S. S. Huang H.-W. C. Schwimmer J. Kim S. Small G. W. (2002). Evaluating early dementia with and without assessment of regional cerebral metabolism by PET: a comparison of predicted costs and benefits. *J. Nuclear Med.* 43 253–266. 11850493

[B56] Skoog I. Davidsson P. Aevarsson O. Vanderstichele H. Vanmechelen E. Blennow K. (2003). Cerebrospinal fluid beta-amyloid 42 is reduced before the onset of sporadic dementia: a population-based study in 85-year-olds. *Dement Geriatr. Cogn. Disord.* 15 169–176. 10.1159/000068478 12584433

[B57] Spiegel J. Pirraglia E. Osorio R. S. Glodzik L. Li Y. Tsui W. (2016). Greater specificity for cerebrospinal fluid P-tau231 over P-tau181 in the differentiation of healthy controls from Alzheimer’s disease. *J. Alzheimers Dis.* 49 93–100. 10.3233/JAD-150167 26444757 PMC4694576

[B58] Stomrud E. Hansson O. Blennow K. Minthon L. Londos E. (2007). Cerebrospinal fluid biomarkers predict decline in subjective cognitive function over 3 years in healthy elderly. *Dement Geriatr. Cogn. Disord.* 24 118–124. 10.1159/000105017 17622715

[B59] Tanyaluck Thientunyakit M. Chakmeedaj Sethanandha M. Weerasak Muangpaisan M. Satoshi Minoshima M. (2021). 3D-SSP analysis for amyloid brain PET imaging using 18F-florbetapir in patients with Alzheimer’s dementia and mild cognitive impairment. *Med. J. Malaysia* 76 493–501. 34305110

[B60] Toledo J. B. Zetterberg H. Van Harten A. C. Glodzik L. Martinez-Lage P. Bocchio-Chiavetto L. (2015). Alzheimer’s disease cerebrospinal fluid biomarker in cognitively normal subjects. *Brain* 138 2701–2715. 26220940 10.1093/brain/awv199 PMC4643624

[B61] Tzourio-Mazoyer N. Landeau B. Papathanassiou D. Crivello F. Etard O. Delcroix N. (2002). Automated anatomical labeling of activations in SPM using a macroscopic anatomical parcellation of the MNI MRI single-subject brain. *Neuroimage* 15 273–289. 10.1006/nimg.2001.0978 11771995

[B62] Vemuri P. Wiste H. Weigand S. Shaw L. Trojanowski J. Weiner M. (2009). MRI and CSF biomarkers in normal, MCI, and AD subjects: diagnostic discrimination and cognitive correlations. *Neurology* 73 287–293. 10.1212/WNL.0b013e3181af79e5 19636048 PMC2715210

[B63] Veronese M. Bodini B. García-Lorenzo D. Battaglini M. Bongarzone S. Comtat C. (2015). Quantification of [11C] PIB PET for imaging myelin in the human brain: a test—retest reproducibility study in high-resolution research tomography. *J. Cereb. Blood Flow Metabol.* 35 1771–1782. 10.1038/jcbfm.2015.120 PMC4635232 26058700

[B64] Yakushev I. Hammers A. Fellgiebel A. Schmidtmann I. Scheurich A. Buchholz H. G. (2009). SPM-based count normalization provides excellent discrimination of mild Alzheimer’s disease and amnestic mild cognitive impairment from healthy aging. *Neuroimage* 44 43–50. 10.1016/j.neuroimage.2008.07.015 18691659

[B65] Zetterberg H. Blennow K. (2021). Moving fluid biomarkers for Alzheimer’s disease from research tools to routine clinical diagnostics. *Mol. Neurodegener* 16:10. 10.1186/s13024-021-00430-x 33608044 PMC7893769

[B66] Zhang K. Mizuma H. Zhang X. Takahashi K. Jin C. Song F. (2021). PET imaging of neural activity, β-amyloid, and tau in normal brain aging. *Eur. J. Nucl. Med. Mol. Imag.* 48 3859–3871. 10.1007/s00259-021-05230-5 33674892

[B67] Ziolko S. K. Weissfeld L. A. Klunk W. E. Mathis C. A. Hoge J. A. Lopresti B. J. (2006). Evaluation of voxel-based methods for the statistical analysis of PIB PET amyloid imaging studies in Alzheimer’s disease. *Neuroimage* 33 94–102. 10.1016/j.neuroimage.2006.05.063 16905334

[B68] Zou K. Abdullah M. Michikawa M. (2020). Current Biomarkers for Alzheimer’s Disease: From CSF to Blood. *J. Pers. Med.* 10:85. 32806668 10.3390/jpm10030085 PMC7564023

