# Supplementary figures and images for: Statistical Parametric Mapping in Amyloid Positron Emission Tomography
Source: Front Aging Neurosci. 2022 Apr 25;14:849932. doi: 10.3389/fnagi.2022.849932 (PMC9083453; doi:10.3389/fnagi.2022.849932)

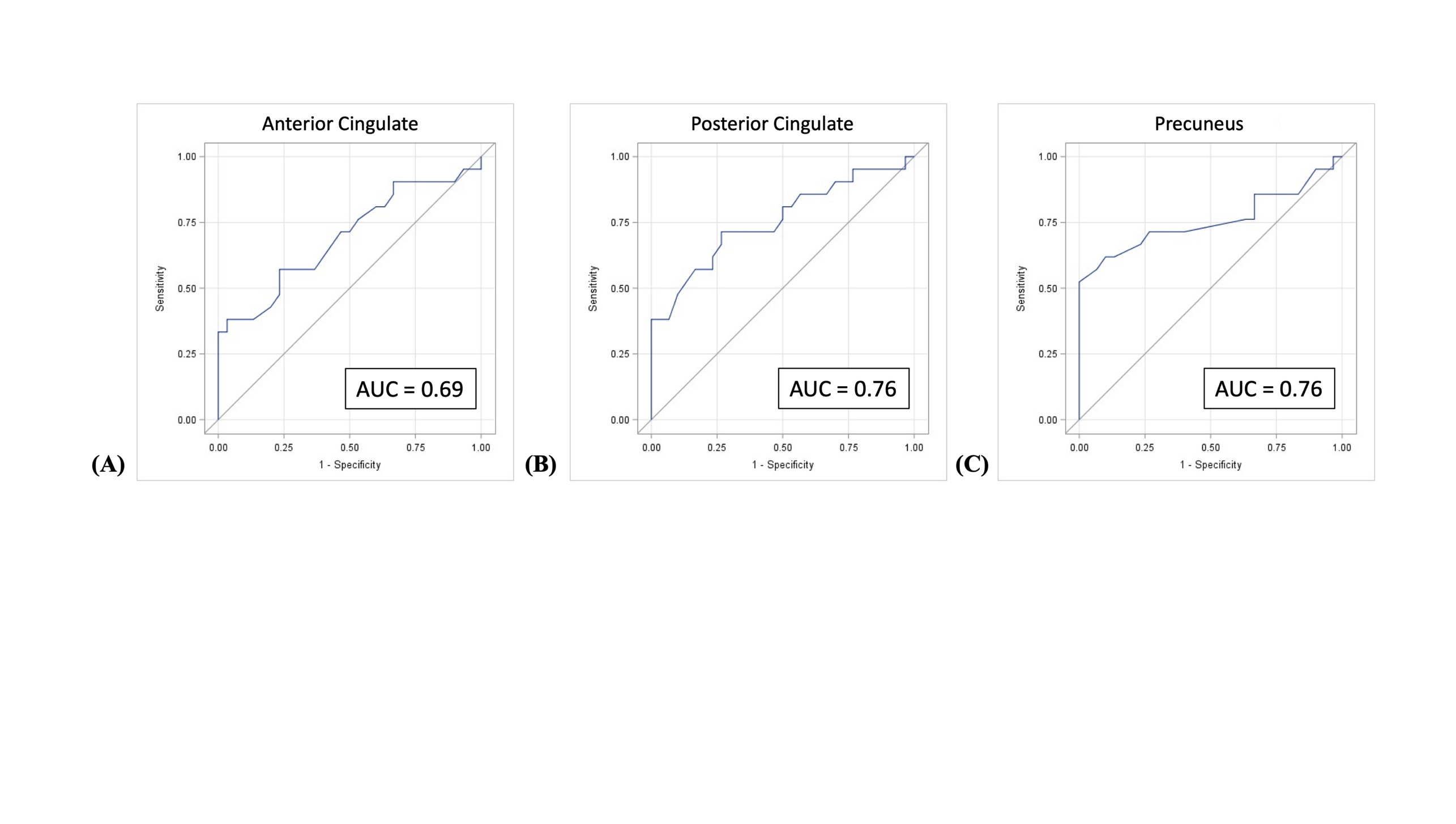

Supplement: Supplementary Figure 1 — Receiver operating characteristic (ROC) curves generated for the anterior cingulate (A), posterior cingulate (B), and precuneus (C) regions. An optimal z-score cutoff for each region was calculated using Youden’s index. [file Image_1.TIFF]
